# The regulatory role of IL-6R in hepatitis B-associated fibrosis and cirrhosis

**DOI:** 10.1590/1414-431X20176246

**Published:** 2017-09-21

**Authors:** Y. Chen, S. Yang, Y. Peng, Z. Yang

**Affiliations:** Department of Infectious Diseases, Linyi People's Hospital, Linyi, Shandong, China

**Keywords:** miRNA-30b, IL-6 receptor, Chronic hepatitis B, Fibrosis, Cirrhosis

## Abstract

This study investigated the expression and regulation of IL-6R in hepatitis B-associated moderate hepatic fibrosis and cirrhosis. Liver tissues, peripheral blood monocytes (PBMs) and serum were collected from 26 hepatitis B patients with liver fibrosis and 35 hepatitis B patients with liver cirrhosis. The levels of *Il-6r* mRNA expression in these samples were examined by quantitative real-time PCR and IL-6R protein levels were analyzed by western blot and ELISA. MiRNAs that regulate IL-6R expression were predicted by bioinformatics analysis, and validated by dual luciferase reporter assay. Compared with the hepatic fibrosis group, IL-6R was significantly upregulated at both mRNA and protein levels in liver tissues, PBMs and serum samples from the hepatic cirrhosis group (P<0.05). The 3′UTR of *Il-6r* mRNA was predicted to contain a miR-30b binding site and IL-6R was identified as a possible target of miR-30b. MiR-30b expression was significantly downregulated in samples from hepatic cirrhosis patients compared with hepatic fibrosis patients (P<0.05). In conclusion, IL-6R was upregulated while miR-30b was decreased in patients with liver cirrhosis. The miR-30 can directly regulate the expression of IL-6R.

## Introduction

A prospective study showed that the annual incidence of cirrhosis developed from chronic hepatitis B is estimated to be 2.1% (1). Another follow-up study on HBeAg negative chronic hepatitis B patients for an average of 9 years (varied from 1 to 18.4 years) showed that the incidence of cirrhosis transition is 23% (1–3). Liver fibrosis was found in most of the chronic liver diseases with different causes, and the morphological characteristic of hepatic fibrosis is a large number of extracellular matrix deposition in the liver tissue (4). With progression, hepatic fibrosis may develop to cirrhosis (5), affecting the health and life of the patient greatly. Therefore, it is highly important for the clinical treatment of hepatitis B to study the mechanism of transition from hepatic fibrosis to cirrhosis at the molecular level.

Hepatitis B is believed to share some characteristics of inflammation (6,7), which may promote the development of hepatic tumor (8,9). As a key factor involved in inflammatory responses, IL-6 was reported to play important roles in the development of hepatitis B (10,11). The formation of IL-6/IL-6 receptor (IL-6R) complex and its subsequent binding to gp130 is indispensable for the biological activity of IL-6 (12). However, the control of IL-6R in hepatitis B-associated liver fibrosis and cirrhosis and the regulatory pathway of IL-6R in this process has yet to be fully understood.

A micro RNA (miRNA) is a small non-coding RNA molecule widely present in eukaryotes with the length of 18-22 nt that regulate protein expression at mRNA level (13–15). It has been found that the expression of many miRNAs and their related proteins was altered during the process of hepatitis B-associated liver fibrosis and cirrhosis (16,17), suggesting that miRNAs may exert critical roles in the regulation of fibrosis- and cirrhosis-related protein expression.

In this study, real-time PCR, western blot and ELISA were used to detect the expression of IL-6R in liver tissues, peripheral blood monocytes (PBMs) and serum from hepatitis B patients with moderate hepatic fibrosis or moderate hepatic cirrhosis. Bioinformatics prediction and dual-luciferase reporter assay were used to determine whether miR-30b can regulate IL-6R.

## Material and Methods

### Patients

A total of 26 cases of hepatitis B patients with moderate liver fibrosis and 35 cases of hepatitis B patients with moderate liver cirrhosis who were diagnosed between August 2013 and January 2016 at the Linyi People's Hospital were enrolled. Among the hepatitis fibrosis group, there were 16 male and 10 female patients, and the median age was 46 years (ranging from 19 to 65 years). Among the hepatitis cirrhosis group, there were 15 male and 20 female patients, and the median age of the patients was 48 years (ranging from 18 to 66 years). All subjects were first diagnosed and confirmed by serological detection, B-ultrasound and liver puncture, and had a negative history of diabetes, tumors or other immunological diseases. Written informed consent was obtained from every patient and the study was approved by the ethics review board of the Linyi People's Hospital.

### Sample collection

Liver tissues of 1.0-1.5 cm length were collected by Color Doppler ultrasound guided ejection biopsy gun. A part of the tissues was stored in liquid nitrogen until analysis and the other part was fixed in 10% formaldehyde solution for H&E and Masson staining. Patients with hepatic fibrosis or hepatic cirrhosis were confirmed according to the diagnostic criteria in "chronic hepatitis B prevention and control guidelines" developed by the Chinese Medical Association, liver disease and infectious disease branch (18).

Serum and peripheral blood monocytes were collected from peripheral blood. In general, 10-15 mL of peripheral blood was placed in 4°C for 1-2 h, and serum in the upper layer was aspirated and centrifuged at 400 *g* for 10 min at 4^o^C. Serum was aliquoted and stored at -80°C. Anticoagulant-treated blood was diluted with an equal volume of Iscove’s modified Dulbecco’s medium (Invitrogen, USA). Eight milliliter diluted blood was layered over 5 mL of Ficoll-Hypaque in a sterile 15-mL conical tube by dribbling slowly down the side of the tube using a pipete. After centrifuging at 400 *g* for 30 min at room temperature, the white cell layer containing the mononuclear cells was in the middle layer. The white cells were removed and placed in a sterile 15-mL polypropylene tube. Cells were then washed twice with 5 volumes of D-Hank's and centrifuged at 300 *g* for 10 min at room temperature. Cells were resuspended into 1×10^6^ cells/mL, and 3×10^6^ cells were inoculated into 9 cm^2^ plates followed by culture for 1-2 h at 37°C. Adherent cells were PBMs.

### RNA extraction and quantitative real-time PCR

Total RNAs were isolated using TRIzol¯ isolation reagent (10606ES60, Yusheng Biotechnology Co., Ltd., China) according to the manufacturer's instructions. Serum RNA was extracted by miRNeasy Serum/Plasma Kit (JL217184, Jianlun Biotechnology, China). After quantification under spectrophotometer, RNA (1 µg) was reverse-transcribed using a TIANScriptIIcDNA 1st strand cDNA Synthesis Kit (Tiangen Biotech Co., Ltd., China) into cDNA. MiRNA was reverse transcribed using miRcute miRNA cDNA Synthesis Kit (Tiangen). Quantitative real-time PCR was performed using the SuperReal PreMix (SYBR Green; Tiangen) for mRNA, or miRcute miRNA qPCR Detection kit (Tiangen) for miRNA. For mRNA quantification, the reaction mixture was incubated for 1 cycle at 95°C for 30 s, followed by 39 cycles at 95°C for 5 s, and 60°C for 20 s. For miRNA, the reaction mixture was incubated for 1 cycle at 95°C for 5 min, followed by 40 cycles at 95°C for 10 s, 60°C for 20 s, and 72°C for 10 s. Primers used were as follows: IL-6R, forward: 5′-TGGTGGATGTTCCCCCCGAG-3′, reverse: 5′-TCCTGGGAATACTGGCACGG-3′; GAPDH, forward: 5′-AAGGCTGTGGGCAAGG-3′, reverse: 5′-TGGAGGAGTGGGTGTCG-3′; miR-30b, forward: 5′-CGCGCTGTAAACATCCTACAC-3′, reverse: 5′-GTGCAGGGTCCGAGGT-3′; U6, forward: 5′-GCTTCGGCAGCACATATACTAAAAT-3′, reverse: 5′- CGCTTCACGAATTTGCGTGTCAT-3′. Rrelative expression was evaluated using the 2^−ΔΔCt^ method. The expression of β-actin and small nuclear U6 was used as the internal control for mRNA and miRNA, respectively.

### Western blot

Proteins were extracted by incubation with RIPA buffer and protease inhibitor PMSF. Protein concentration was determined by using BCA assay kit (RTP7102, Real-Times Biotechnology Co., Ltd., China). Then, 20 µg of proteins were separated on 10% SDS-Page and transferred to polyvinylidene difluoride membranes. After blocking with 5% skimmed milk for 1 h, the membranes were probed with the following antibodies: rabbit anti-IL-6R (1:1000, ab128008, Abcam, USA) or rabbit anti-β-actin (1:5000, ab129348, Abcam) at 4°C overnight. For detection, goat anti-rabbit (1:3000, ab6721, Abcam) secondary antibodies conjugated to horseradish fluoride were used. Signal detection was performed using chemiluminescence reaction (ECL; ab65623, Abcam). The acquired images were analyzed by Image Lab 3.0 (Bio-Rad Laboratories, USA) and the relative protein expression is reported as the densitometric value ratio of IL-6R band to β-actin band.

### ELISA

Il-6R protein levels in serum were measured by IL-6R ELISA kit (QY-PF9440, Qiaoyu Biotechnology Co., Ltd., China) according to the manufacturer's instruction. In brief, 1:4 dilution of serum samples and eight serial dilutions of standard substrate at the volume of 50 µL were incubated overnight at 4°C. After horseradish-peroxidase conjugated secondary antibody incubation for 1 h followed by washing for 5-times, the chromogenic substrate solution was added and the reaction was stopped with H_2_SO_4_ and read at 450 nm.

### Bioinformatics analysis

To further identify the miRNAs that may regulate the expression of Il-6R, five bioinformatics software, including miRanda (http://www.microma.org/rnicroma/home.do), TargetSean (www.targetscan.org), PiTa (http://genie.weizmann.ac.il/pubs/mir07/mir07_data.html), RNAhybrid (http://bibiserv.techfak.uni-bielefeld.de/rnahybrid/) and PICTA (http://pictar.mdc-berlin.de/) were used.

### Dual-luciferase reporter gene assay

According to the results of bioinformatics prediction, the conservative miR-30b binding sequence on 3′UTR of *Il-6r* mRNA (wildtype) or the mutant sequence was cloned. Luciferase reporter plasmids were generated by insertion of wild type or mutant sequences of Il-6r into the multiple cloning site (Spe-1 and *Hind*III) downstream of the luciferase reporter gene in the pMIR-REPORT™ Luciferase kit (ThermoFisher Scientific, USA). HEK293T cells were transfected with 0.8 μg constructed luciferase reporters and 100 nM agomiR-30b or negative control RNA (NC). A total of 10 ng pMIR-REPORT™ β-gal Control Plasmid was transfected as an internal control for transfection efficiency. Luminescence was measured at 24 h after transfection using Dual-Luciferase¯ Reporter Assay System (Promega, USA) according to the manufacturer's instructions. Measurements of luminescence were performed on Glomax 20/20 (Promega).

### Statistical analysis

Data analysis was carried out using the SPSS 18.0 (IBM Corp., USA) and reported as means±SD. Normality test was performed. Differences between groups were evaluated for significance using one-way ANOVA. LSD or SNK methods were used when variances were equal, and Tamhane's T2 or Dunnett's T3 methods were used when variances were unequal. P<0.05 was considered to be statistically significant.

## Results

### *Il-6r* mRNA expression in liver tissues, PBMs and serum

**Figure 1.**
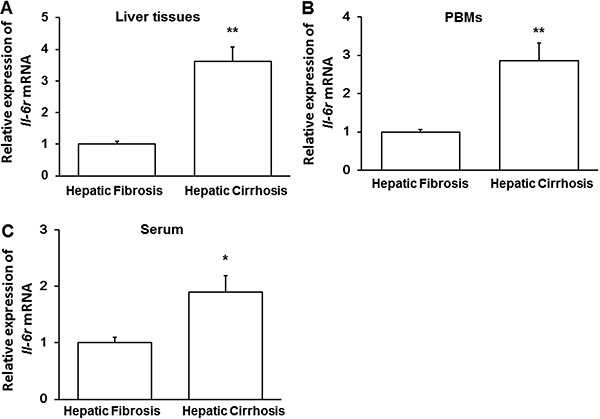
Real-time PCR analysis of *Il-6r* mRNA expression. *A*, Liver tissues; *B*, peripheral blood monocytes (PBMs), and *C*, serum from hepatitis B patients with liver fibrosis or liver. Data are reported as means±SD. *P<0.05 and **P<0.01, one-way ANOVA.

*Il-6r* mRNA expression was significantly increased in hepatic cirrhosis samples compared with hepatic fibrosis samples (P<0.05, Figure 1), suggesting that IL-6R is elevated in liver cirrhosis patients.

### IL-6R protein expression in liver tissues, PBMs and serum

**Figure 2.**
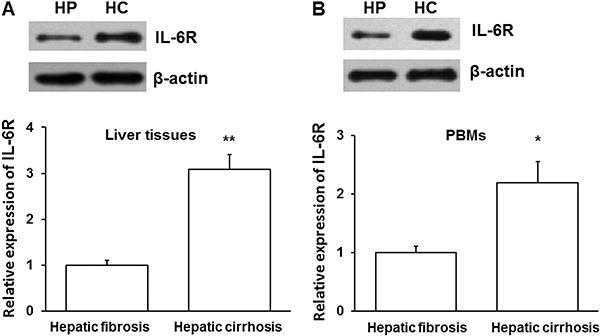
Relative protein expression by western blot analysis of IL-6R protein expression. *A*, Liver tissues, and *B*, peripheral blood monocytes (PBMs) from hepatitis B patients with liver fibrosis (HP) or liver cirrhosis (HC). β-actin expression was detected as internal control. Data are reported as means±SD. *P<0.05 and **P<0.01, one-way ANOVA.

**Figure 3.**
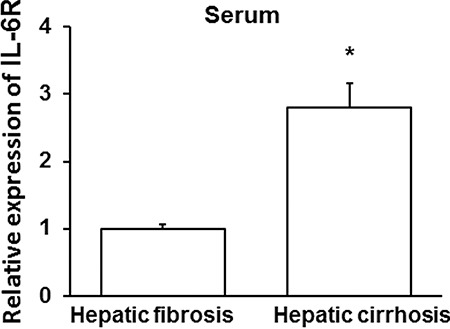
ELISA analysis of IL-6R protein expression in serum from hepatitis B patients with liver fibrosis and liver cirrhosis. Data are reported as means±SD. *P<0.05, ANOVA.

As expected, IL-6R protein level was also upregulated in patients with cirrhosis compared with patients with hepatic fibrosis (P<0.05, Figures 2 and 3), indicating that upregulated IL-6R expression may regulate the development of hepatic cirrhosis in hepatitis B patients. In addition, since IL-6R in serum mainly comes from PBMs, the increased IL-6R levels in serum may be induced by the higher IL-6R in PBMs from hepatic cirrhosis patients.

### miR-30b targeted IL-6R 3′UTR

**Figure 4.**
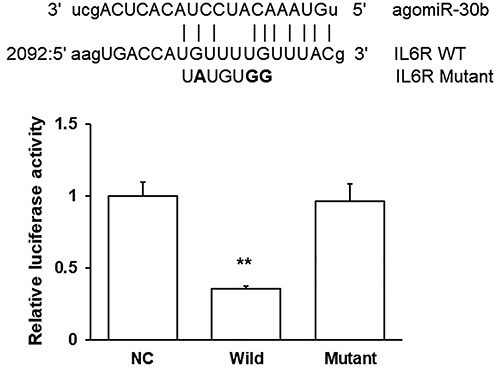
IL-6R is a possible target of miR-30b. The wildtype (WT) and mutant *Il-6r* 3′UTR luciferase reporter constructs were co-transfected with negative control RNA (NC) or agomiR-30b, respectively. Luciferase activities were assayed 24 h post transfection. Data are reported as means±SD. **P<0.01, compared with NC (one-way ANOVA).

A miR-30b binding site was found at the 3′UTR of *Il-6r* mRNA. Dual luciferase reporter assay was performed to confirm this prediction. Co-transfection of agomiR-30b and pMIR-REPORT-IL-6R wild type construct significantly decreased luciferase activity (P<0.05) compared with co-transfection of NC and pMIR-REPORT-IL-6R wild type construct. However, agomiR-30b and pMIR-REPORT-IL-6R mutant construct co-transfection did not reduce luciferase activity (Figure 4). These results suggest that IL-6R is a possible target of miR-30b through the binding of miR-30b to its 3′UTR sequences.

### Expression of miR-30b in hepatic fibrosis and hepatic cirrhosis samples

**Figure 5.**
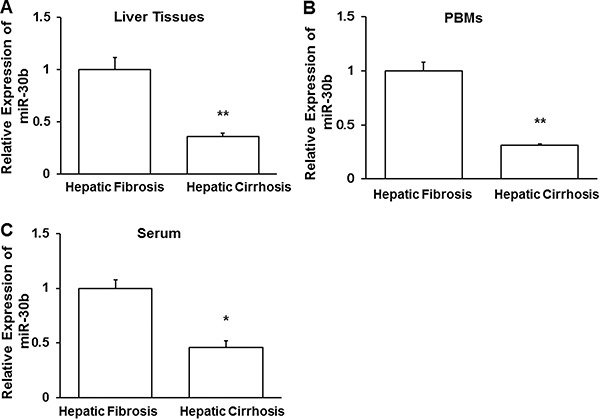
Real-time PCR analysis of miR-30b expression in *A*, liver tissues, *B*, peripheral blood monocytes (PBMs), and *C*, serum from hepatitis B patients with liver fibrosis or liver cirrhosis. Data are reported as means±SD. *P<0.05 and **P<0.01, one-way ANOVA.

As shown in Figure 5, miR-30b expression was significantly downregulated in samples from patients with hepatic cirrhosis compared with those with hepatic fibrosis. The negative correlation between IL-6R and miR-30b in patients indicates that miR-30b may regulate IL-6R expression in hepatic cirrhosis.

## Discussion

In this study, we investigated the expression of IL-6R as well as its possible regulator miR-30b in hepatitis B patients with moderate liver fibrosis or moderate liver cirrhosis. The liver tissues, PBMs and serum were collected.

IL-6, which is upregulated in immune responses stimulated by bacteria, endotoxin, dust particles and other exogenous particles is a multi-functional cytokine (19). It exerts important functions in immune responses, inflammation, cell differentiation, coagulation and tumor development, and its expression is significantly upregulated in inflammatory responses caused by damage, trauma, stress, infection and other factors (20). IL-6 can induce the body to produce C-reactive protein and fibrinogen, and can promote thrombosis in inflammation (21). Increased IL-6 in the body could induce the development of inflammatory diseases, such as rheumatoid arthritis and Crohn's disease, by its binding to IL-6R (22). In rheumatoid arthritis, IL-6 stimulates the production of inflammatory cytokines by T and B lymphoid cells, promotes the maturation and differentiation of B cells, and enhances the effects of IL-1β and TNFα (23). In addition, IL-6 has chemotactic function for other inflammatory cells including neutrophils and monocyte/macrophages (24). In summary, these studies show the important role of IL-6 in inflammatory responses. To exert its activity, IL-6 needs to bind to membrane receptor IL-6R that is restrictedly expressed on hepatic cells, monocytes/macrophages and certain lymphoid cells, and the IL-6/IL-6R complex subsequently combines with gp130 to activate cell signaling through gp130 dimerization (25).

Here we found upregulated IL-6R mRNA and protein expression in liver tissues, PBMs and serum from hepatitis B-associated cirrhosis patients compared with hepatitis B associated fibrosis patients. IL-6R upregulation could be a result of monocyte and lymphocyte activation since these cells secret abundant IL-6 in antigen-induced immune response. Activation of these cells and IL-6 secretion is also a cause of cell damage. However, the role and mechanism of IL-6R upregulation in the transition of liver fibrosis to cirrhosis still needs further investigation.

The regulation of mRNA transcription and expression is a complex process involving many factors (26). To investigate the upstream regulation of IL-6R, we focused on a group of miRNAs. These miRNAs negatively regulate their target mRNA by binding to the 3′UTR of mRNA to repress their expression, therefore function as key regulators in development, physiology and diseases. Some miRNAs have been identified as disease biomarkers (27). By using bioinformatics methods, we assessed miRNAs that lie upstream of IL-6R, and found that miR-30b may regulate IL-6R expression. miR-30b is a member of the miR-30 family, which includes five miRNAs, miR-30a-e (28). These five miRNAs come from different chromosomes and are allocated to a family due to the homology of their seed sequence.

Many studies have demonstrated the role of miR-30b in inflammatory responses, tumor malignance and epithelial mesenchymal transition (29–32). Additionally, miR-30b has positive effects on nerve repair, apoptosis repression and vascular regeneration (33–37). For example, miR-30b regulates the normal and tumorigenic development of human neural tube cells whereas aberrantly expressed miR-30b expression may result in schizophrenia (33). GalNAc transferases regulated by miR-30b enhances invasion and immunosuppression during metastasis (34). In breast cancer, trastuzumab inhibits cancer cell proliferation by upregulating miR-30b (35). Moreover, miR-30b also plays roles in the reproductive system, by regulating mammary gland development and involution or endometrial receptivity (36,37). These studies have proven that miR-30b is closely related to cell growth, development, differentiation and migration. Our results are in line with these previous reports. First, expression of miR-30b was downregulated whereas IL-6R was upregulated in liver tissues and PBMs from hepatitis B associated-cirrhosis patients compared with hepatitis B-associated fibrosis patients, indicating that miR-30b negatively regulated IL-6R expression. Second, a similar opposite expression pattern of miR-30b and IL-6R was also observed in serum, suggesting that downregulation of miR-30b in PMCs leads to higher IL-6R in serum.

In conclusion, IL-6R was increased whereas miR-30b was decreased in patients with liver cirrhosis. Furthermore, miR-30b could directly regulate the expression of IL-6R. Further studies are needed to investigate the role and mechanism of IL-6R and miR-30b in the transition of liver fibrosis to cirrhosis.
